# Who is your prenatal care provider? An algorithm to identify the predominant prenatal care provider with claims data

**DOI:** 10.1186/s12913-024-11080-2

**Published:** 2024-05-27

**Authors:** Songyuan Deng, Samantha Renaud, Kevin J. Bennett

**Affiliations:** University of South Carolina School of Medicine, Columbia, SC USA

**Keywords:** Prenatal care, Predominant provider, Visit frequency, Visit sequence

## Abstract

**Background:**

Using claims data to identify a predominant prenatal care (PNC) provider is not always straightforward, but it is essential for assessing access, cost, and outcomes. Previous algorithms applied plurality (providing the most visits) and majority (providing majority of visits) to identify the predominant provider in primary care setting, but they lacked visit sequence information. This study proposes an algorithm that includes both PNC frequency and sequence information to identify the predominant provider and estimates the percentage of identified predominant providers. Additionally, differences in travel distances to the predominant and nearest provider are compared.

**Methods:**

The dataset used for this study consisted of 108,441 live births and 2,155,076 associated South Carolina Medicaid claims from 2015–2018. Analysis focused on patients who were continuously enrolled throughout their pregnancy and had any PNC visit, resulting in 32,609 pregnancies. PNC visits were identified with diagnosis and procedure codes and specialty within the estimated gestational age.

To classify PNC providers, seven subgroups were created based on PNC frequency and sequence information. The algorithm was developed by considering both the frequency and sequence information. Percentage of identified predominant providers was reported. *Chi*-square tests were conducted to assess whether the probability of being identified as a predominant provider for a specific subgroup differed from that of the reference group (who provided majority of all PNC). Paired *t*-tests were used to examine differences in travel distance.

**Results:**

Pregnancies in the sample had an average of 7.86 PNC visits. Fewer than 30% of the sample had an exclusive provider. By applying PNC frequency information, a predominant provider can be identified for 81% of pregnancies. After adding sequential information, a predominant provider can be identified for 92% of pregnancies. Distance was significantly longer for pregnant individuals traveling to the identified predominant provider (an average of 5 miles) than to the nearest provider.

**Conclusions:**

Inclusion of PNC sequential information in the algorithm has increased the proportion of identifiable predominant providers by 11%. Applying this algorithm reveals a longer distance for pregnant individuals travelling to their predominant provider than to the nearest provider.

**Supplementary Information:**

The online version contains supplementary material available at 10.1186/s12913-024-11080-2.

## Background

Having a usual source of care has a substantial positive impact on patients and outcomes. Such continuity of care (COC), due to the usual source of care [1, 2], is associated with reduced avoidable hospitalization and emergency department visits [3, 4], cost reduction [4, 5], and reduced probability of mortality [6, 7]. Thus, being able to identify a patient’s usual source of care or where they predominantly receive care is important for healthcare resource planning for providers and policymakers.

In the 1990s, algorithms were introduced that identified the most frequently visited providers, known as plurality providers (a provider who provides the most visits), and the majority provider (a provider who provides majority of visits), using claims data. The majority provider is a subset of the plurality provider because a majority provider is by definition a plurality provider, but a plurality provider is not necessarily a majority provider [8, 9]. These algorithms used the proportion of visit frequency during a period as well as the specialty of a provider. They have been applied to describe what percentage of care was delivered by an identified predominant provider [10] and as measures of care coordination [11]. Recently these algorithms, designed to utilize claims data, have been verified via electronic health records. The results showed that in primary care settings, these algorithms identified the predominant providers for 78–84% of primary care physician visits but only 25–56% for all visits. Due to the fact that the majority provider is a subset of the plurality provider, the plurality algorithm identified the predominant providers for 84% of primary care physician visits, but the majority algorithm identified only 78% [12].

This plurality algorithm has been applied outside of the primary care setting for patients seeking care for dementia [13] and for those seeking care for HIV [14]. Based on this literature review, no study was identified that applied this algorithm to identify the predominant provider for prenatal care (PNC), despite pregnancy complications (47%) and the associated cost [15]. Furthermore, Kotelchuck proposed the Adequacy of Prenatal Care Utilization (APNCU) Index specifically for PNC, [16] which has since been widely adopted. The APNCU index integrated the adequacy of initiation of PNC and the adequacy of received services. Therefore, to identify the predominant provider for PNC, an algorithm needs to include both the PNC visit frequency and sequence information, such as the first and last visits. Such visit sequential information was not included in previously proposed algorithms.

Furthermore, previous algorithms could not identify the predominant providers for 16–25% of the studied population [12]. Public programs aiming to increase prenatal care access /capacity need provider information to tailor interventions to specific areas. By integrating sequence with frequency information, this study aims to identify more predominant providers than previous algorithms. The increased proportion of identifiable predominant providers can provide supportive information for these public programs.

This study constructed a version of the plurality provider algorithm specifically for PNC by including both the visit frequency and sequence information. This algorithm was applied to pregnant individuals to identify their predominant providers. As PNC frequency varies, different patterns were adopted to compare the final percentage of pregnant individuals whose predominant providers were identified. Finally, this study examined the differences between the travel distance to the nearest visited provider and that to the predominant provider because long distance is one of the main factors that limit access to PNC [17].

## Methods

### Data and sample

This study utilized deidentified Medicaid claims data from the South Carolina Revenue and Fiscal Affairs (SCRFA) Office for 2014–2019. Medical claims were acquired for those who had a live birth during the period 2015–2019 in the 12 months prior to delivery. Vital records were used to identify live births with a parent-baby linkage by the SCRFA office. Exemption for this study was obtained from the Institutional Review Board at the authors’ institute due to the secondary analysis using deidentifiable administrative data.

A total of 108,441 live births, with 2,155,076 associated claims, were found during the 2015–2018 period. Full date of birth (DOB) was not released due to privacy concerns. However, as allowed by our data use agreement, and using previously published algorithms as guidance, [18, 19] month of birth was estimated by using the dates of service and International Classification of Diseases (ICD) associated with the claims and appended to SC RFA provided year of birth. Samples were delimited to those who had continuous enrollment in Medicaid for the duration of their pregnancies, resulting in 36,848 pregnancies. Among those, only 32,609 included at least one prenatal care claim in the data. Analyses were conducted at the pregnancy level because the purpose is to identify a predominant provider for each pregnancy episode.

### Observed prenatal care

This study employed a series of variables to identify PNC visits from these claims. These variables included date of delivery (month/year); date of services (month/year); primary/secondary diagnosis (ICD 9/10 clinical modification (CM), Healthcare Common Procedure Coding System (HCPCS) and Current Procedural Terminology (CPT); and provider specialty.

Office claims dataset was linked to Vital Statistics and hospital claims dataset to construct pregnancy episodes [19]. Delivery date and services date were used to calculate the duration from each claim to delivery, guided by the previously published algorithm [19]. Claims within the gestational age were kept. PNC services were identified with procedure code, including CPT and HCPCS code, and primary diagnosis code. Details can be found in Appendix Table 1.

**Table 1:** Total prenatal care visits and at pregnancy and the specialty-specified provider level

	Pregnancy	Providers
Provider Counts	N/A	1,922
N	32,609	93,712
PNC Mean	7.86	2.73
Standard deviation	4.12	2.71
PNC > 14 (%)	4.62	0.50
14 ≥ PNC > 8 (%)	40.22	5.07
8 ≥ PNC > 1(%)	48.99	48.51
PNC = 1 (%)	6.18	45.92

*N/A*-not applicable

Claims were selected by Medicaid provider specialty and type code, including midwife (06), primary care physician (12,14,19,78), Obstetrics and Gynecology specialist (16, 26, 27), organization (50-FQHC, 97-RHC), nurse practitioner (86), others (02, 10, 40, 48, 57, 94, 95, PA, missing). Specialties that were marked as missing and the supervisor’s specialties of PA (physician assistants) were unknown (0.24%). Although they only accounted for 0.05% of visits, specialties with codes of 02, 10, 40, 48, 57, 94 and 95 were kept for three reasons. First, a coded PNC visit to these specialties satisfies the three criteria of COC [20]. Second, those specialties may serve as a substitute for a traditional PNC provider given the limited access to PNC providers for some populations [21, 22]. Finally, pregnancies diagnosed with hemorrhage during early pregnancy may utilize emergency service (code: 02), those diagnosed with diabetes may consult a diabetes educator (code: 94) for advice on diabetes management during pregnancy, and those diagnosed with depression and anxiety may consult a psychiatrist (code: 48) for medication during pregnancy [15].

This study integrates PNC initiation, frequency of visits, and sequence of visits to inform the algorithm. Visit frequency at the provider level was then used to estimate the PNC fraction, that is, the percentage of PNC visits to a specific provider, given all PNC visits, for a specific pregnancy. Visit sequences were identified using the date of services. The first and last PNC visits were then confirmed with sequential information. PNC initiation refers to the provider that provided the first visit during the pregnancy. The final PNC visit prior to birth plays a particularly important role in ensuring continuity of care during prenatal, delivery, and postpartum care. Some providers may be affiliated with a hospital and capable of providing delivery-related services, while others may not have appropriate training or not be available to provide delivery services and need to refer their patients to a qualified provider [23]. The provider visited at the last PNC visit is considered the referrer for delivery. Therefore, the initial and last PNC visits were identified for each pregnancy.

### Provider definition

**Fig. 1:**
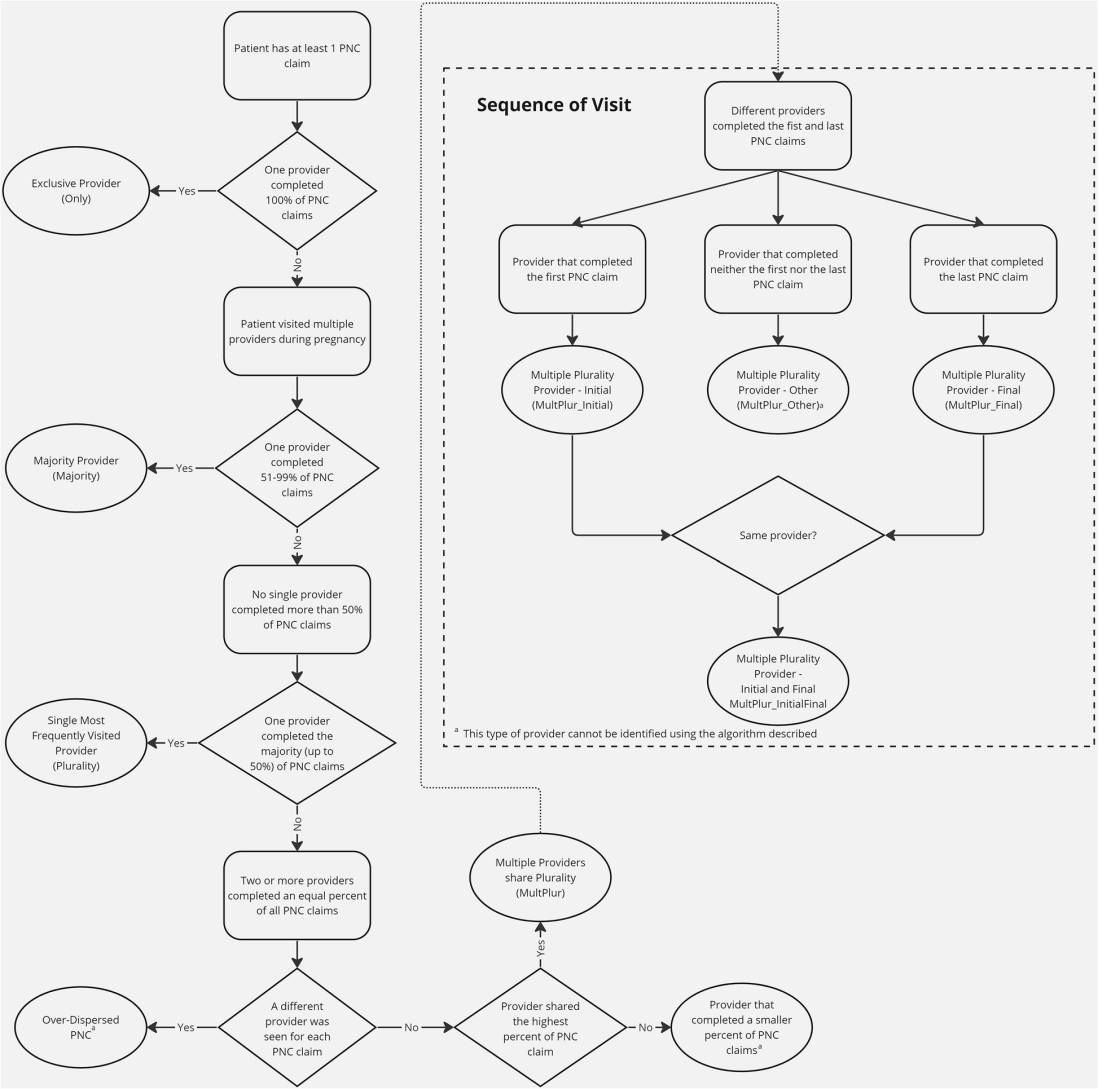
Flowchart of identifying the predominant provider

To identify the predominant provider, this study constructed four categories based on the PNC fraction and sequential information: the exclusive provider (Only), the majority provider (Majority), the single most frequently visited (Plurality) providers, and multiple providers sharing plurality (MultPlur). The PNC fraction was used to measure how many visits a provider served for a given pregnancy, calculated by dividing the number of PNC visits from that specific provider by the total number of PNC visits. Only providers are those who are the only visited provider throughout the pregnancy, with a fraction of 100%. Majority providers are those providing more than half of PNC services for a pregnant individual, with a fraction larger than 50%—Majority provider is aligned with the majority algorithm [8]. Plurality providers are those providing the highest fraction of PNC within all visited provider but less than 50% for a pregnant individual- Plurality provider is aligned with the plurality algorithm [9]. If there were multiple providers who provided equal fractions of PNC visits to a pregnant individual, they were classified as multiple plurality (MultPlur). Providers who were not classified as any of these categories would not be identified as a predominant provider (Fig. 1).

The data obtained from RFA included a unique provider identifier that may refer to a professional or a provider, whichever is applicable, enabling PNC frequency to be counted at the provider identifier level. Since different professionals may share the same provider identifier, frequency can be more accurately counted by combining specialty with this provider identifier. This study used the specialty-specified provider identifier (SSPI) to denote unique providers and calculate PNC frequency.

### Different patterns

This algorithm first counted the PNC frequency at both the pregnancy and provider levels for each pregnancy. The number of PNC providers was also counted for each pregnancy. Every PNC provider was then classified into one of five categories: Only, Majority, Plurality, Multiple Plurality and others. The predominant provider was identified initially as Only through MultPlur providers as shown in Fig. 1. In the initial pattern, the Only providers were identified as the predominant provider. Both Only and Majority providers were identified as the predominant provider in the second pattern. Beyond Only and Majority providers, Plurality providers were identified as the predominant provider in the third pattern. The multiple plurality providers were further included as the predominant provider in the fourth, fifth and sixth patterns. (see Appendix Table 2) The final group of others, including those provide some but not the largest fraction of PNC and MultPlur providers who provide neither the first nor the last PNC.

**Table 2:** The percentage of providers for a pregnant individualat the specialty specified provider level

Providers	Definition	Percentage
PNC provider counts	All PNC providers	2.87
Only	Fraction = 100%	28.40
Majority	Fraction (50%, 100%)	19.55
Plurality	Largest fraction (*N* = 1)	33.49
MultPlur	Largest fraction (*N* > 1)	18.55
Dispersal MultPlur	Largest fraction & COC = 0	7.12
MultPlu_Initial	Largest fraction & 1st visit	7.31
MultPlu_Final	Largest fraction & last visit	3.06
MultPlu_InitialFinal	Largest fraction, 1st & last visit	1.42
2	Largest fraction (*N* = 2)	12.18
3	Largest fraction (*N* = 3)	3.87
4	Largest fraction (*N* = 4)	1.41
5	Largest fraction (*N* = 5)	0.63
6	Largest fraction (*N* = 6)	0.29
7	Largest fraction (*N* = 7)	0.12
8	Largest fraction (*N* = 8)	0.03
9	Largest fraction (*N* = 9)	0.02
10	Largest fraction (*N* = 10)	0.00
11	Largest fraction (*N* = 11)	0.00

*NA* not applicable

Only – The exclusive PNC provider for one pregnancy. Majority – this provider served more than half of all visits for one pregnancy. Plurality – the uniquely most frequently visited provider, who is the only one who served the most visits for a patient (the percentage of this category equals that of MultPlur at count level 1). MultPlur – most frequently visited provider, who served the most visits for a patient. MultPlur_Initial—most frequently visited provider, who served the most visits and the first visit for a patient. MultPlur_Final—most frequently visited provider, who served the most visits and the last visit for a patient. MultPlur_InitialFinal—most frequently visited provider, who served the most visits, first and the last visits for a patient. Dispersal MultPlur: the number of total PNC visits equals the number of total providers. COC: continuity of care index. N: number of providers for a given pregnancy. For example, largest fraction (*N* = 1) means that only one provider has provided all PNC and the fraction equals 100%. ${Fraction}_{i}={n}_{i}\!\left/n\right.$, n is the total number of PNC visits, and ${n}_{i}$ is the number of PNC visits for the *i*th provider

*n* = 32,609

As pregnant individuals may utilize additional providers that differ from the initial provider accessed during their pregnancy [24], different providers may cover different gestation periods for different individuals. For example, some of the most visited providers may serve the early pregnancy period, while others may serve later terms. In the early visit (fourth) pattern, the MultPlur provider which conducts the initial visit is defined as MultPlur_Initial. In the late visit (fifth) pattern, the MultPlur provider that conducts the final visit is defined as MultPlur_Final. In the hybrid (sixth) pattern, the MultPlur provides the initial or the final visits (Fig. 1). Therefore, MultPlur providers in different patterns may provide care for different needs. The MultPlur_Initial in the early patternmay be associated with PNC initiation, while the MultPlur_Final in the late scenario may be associated with actual delivery. The MultPlur in the hybrid patternmay be associated with either or both needs. If there is a tie between MultPlur_Initial and MultPlur_Final, this algorithm gave priority to MultPlur_Initial because APNCU listed PNC initiation as one major measurement [16]. The algorithm developed here will include all three patterns for the MultPlur providers. The percentage of pregnant individuals with a predominant provider was reported for all six patterns.

The percentage of pregnancies with identifiable predominant prenatal care providers by prenatal care frequency were then summarized. *Chi-square* tests were applied to examine whether there is a difference between each category of providers and Majority providers in being identified as a predominant provider, given the total number of PNC visits.

A special case occurs when a pregnant person visits a different provider at each PNC visit. That means that the number of total visits equals the number of providers. This group is defined as over-dispersed (Fig. 1). The dispersed nature of all visits makes the first and last visits contain no meaningful sequential information. The predominant provider cannot be reasonably identified under such a situation. Predominant providers could not be identified for those pregnancies.

### Application example with travel distance

The results of this algorithm were then applied to examine travel distance differences between the nearest PNC provider and the identified predominant PNC provider. For simplicity, this was delimited to visits that occurred within the state boundaries. Any provider who delivered at least one PNC service in this study was included in the analysis. Distance was estimated using Google Maps, which calculated the distance from the pregnant person’s zip-code of residence to the provider’s billing zip-code, measured as centroid to centroid. This study conducted crossworking of zip codes to ZCTAs (Zip Code Tabulation Area). In cases where the zip-codes were the same, the radius of the zip-code area was estimated using the formula area = *π* r^2^, where the zip-code area was acquired from the 2010 census. A provider with the shortest distance for each patient was identified as the nearest provider. A paired *t* test was used to compare the travel distances between the two provider types. All analyses were performed using SAS software version 9.4 (SAS Institute Inc., Cary, NC) at a significance level of 95%.

## Results

Table 1 summarizes the total number of PNC visits for 32,609 pregnancies, and PNC visits at 1,922 providers, resulting a total number of 93,712 pregnancy-provider records. The average total number of PNC visits for all included samples was 7.86. At the pregnancy-provider level, the average number of PNC visits was 2.73. The number of total PNC visits was divided into four categories based on expected PNC frequencies and those with only one PNC visit. Of all pregnancies, 4.62% had more than 14 visits, 40.22% had 9–14 visits, 48.99% had 2–8 visits, and 6.18% had only one visit. At the provider level, however, most providers provided less than 9 times of PNC (8 ≥ PNC: 94.93%).

Table 2 summarizes the number of PNC providers of different definitions at various levels. The average number of providers per person was 2.87, with slightly more than 7% of pregnant individuals experiencing extremely over-dispersed PNC visits (Fig. 1). Less than 30% of all pregnant individuals in this sample exclusively visited an Only provider. Another 20% of all pregnant individuals had more than half of their visits with a Majority provider. Additionally, one-third of all pregnant individuals had most of their visits with a Plurality provider. For 7.35% of pregnant individuals, the MultPlur_Initial provided the first PNC visit; for 8.31% of pregnant individuals, the MultPlur_Final provided the last PNC visit; and for 1.43% of pregnant individuals, a single MultPlur_InitialFinal provided both the first and the last PNC visits.

Table 3 presents the percentage of predominant PNC providers, subset by PNC frequency. For all pregnant individuals, the predominant provider was identified mostly as the order of Plurality, Only, Majority, MultPlur_Initial, and MultPlur_Final. Compared with a Majority provider, pregnant individuals who received 2 ~ 8 times PNC were more likely to be identified with a MultPlur_Initial or no identifiable predominant provider (both *p* < 0.001) and less likely to be with a Only (*p* < 0.001) or MultPlur_Final (*p* = 0.014). Compared with a Majority provider, pregnant individuals who received 9 ~ 14 times PNC were more likely to be identified with a MultPlur_Final (*p* = 0.001) and less likely to be with a Only provider, MultPlur_Initial, or no identifiable predominant provider (all *p* < 0.001). Compared with a Majority provider, pregnant individuals who received more than 14 times PNC were more likely to be identified with a MultPlur_Initial (*p* < 0.001) and less likely to be with a Only provider (*p* = 0.004) or no identifiable predominant provider (*p* < 0.001). Table 4 summarizes the final percentage of predominant PNC providers in different patterns based on the results from Table 3. The final percentage ranged between 28.4% and 81.4%, with only frequency information. After integrating sequential information, the percentage ranged between 88.5% and 91.8%.

**Table 3:** The percentage of pregnancies with identifiable predominant prenatal care providers by prenatal care frequency

Prenatal care frequency	All providers	Only	Majority (ref)	Plurality	MultPlur_Initial	MultPlur_Final	MultPlur_InitialFinal	Dispersal MultPlur
All frequency	100.00	28.40	19.55	33.49	7.31	3.05	1.42	8.19
PNC = 1	6.18	6.18	0.00	0.00	0.00	0.00	0.00	0.00
8 ≥ PNC > 1	48.99	12.24^***^	9.01	14.98	4.08^***^	1.28^*^	0.70	7.40^***^
14 ≥ PNC > 8	40.22	8.78^***^	9.52	16.52	3.03^***^	1.66^*^	0.67	0.70^***^
PNC > 14	4.62	1.20^**^	1.02	1.99^***^	0.20	0.12^**^	0.05	0.09^***^

Only – The exclusive PNC provider for one pregnancy. Majority – this provider served more than half of all visits for one pregnancy. Plurality – the uniquely most frequently visited provider, who is the only one who served the most visits for a patient (the percentage of this category equals that of MultPlur at count level 1). MultPlur_Initial—most frequently visited provider, who served the most visits and the first visit for a patient. MultPlur_Final—most frequently visited provider, who served the most visits and the last visit for a patient. MultPlur_InitialFinal—most frequently visited provider, who served the most visits, first and the last visits for a patient. Dispersal MultPlur: the number of total PNC visits equals the number of total providers. *Chi-*square tests were applied to this Table and Majority was the reference group

^*^p < .05

^**^p < .01

^***^p < .001

**Table 4:** The final percentage of identifiable predominant providers in different patterns

Patterns (providers)	Percentage
1, Only	28.40
2, Only and Majority	47.95
3, Only Majority and Plurality	81.44
4, Only Majority, Plurality and MultPlur_Initial	88.75
5, Only, Majority, Plurality and MultPlur_Final	88.40
6, Only, Majority, Plurality and MultPlur_Initial or MultPlur_Final	91.81

Only – The exclusive PNC provider for one pregnancy. Majority – this provider served more than half of all visits for one pregnancy. Plurality – the uniquely most frequently visited provider, who is the only one who served the most visits for a patient (the percentage of this category equals that of MultPlur at count level 1). MultPlur– most frequently visited provider, who served the most visits for a patient. MultPlur_Initial—most frequently visited provider, who served the most visits and the first visit for a patient. MultPlur_Final—most frequently visited provider, who served the most visits and the last visit for a patient

This algorithm was then applied to measure the travel distance from pregnant individuals to providers using the sixth pattern. The travel distances were compared within the South Carolina boundary, and the sample size was reduced to 29,763 (91.3%). The distance was significantly shorter (19.3 vs. 24.2 miles) for pregnant individuals traveling to the nearest visited PNC provider than to the predominant PNC provider (mean difference:—4.8 miles, 95% Confidence interval:—4.6—-5.1 miles, *p* < 0.0001).

## Discussion

This study developed a practical algorithm to identify the predominant PNC provider based on guidelines and utilization patterns. The final percentages of identifiable predominant PNC providers were reported based on various patterns. Applying prior algorithms, this study only identified approximately 81% of pregnancies with a predominant provider. After adding sequential information, approximately 92% of pregnancies can now be identified. Except for 7% of over-dispersed cases, only 1% of included pregnancies could not be identified with a predominant provider using this algorithm. Applying this algorithm revealed that pregnant individuals travel a longer distance to their predominant PNC provider than to the nearest visited PNC provider.

The implications for this algorithm are threefold. First, the algorithm integrates visit frequency and sequence information, rather than relying solely on frequency as in previous algorithms [8, 9]. A recent study applied three different criteria when both plurality and majority algorithms fail. If two providers were equally eligible to be listed as the predominant provider, the study used sequence (the last visit), expenditure and duration to exclude one of them, resulting in one predominant provider [12]. In this study, the well-accepted APNCU was utilized [16] as a justification for including sequence information into the proposed algorithm. Therefore, the percentage of identified predominant providers increased from 81% in the third pattern to 88% in the fifth pattern and to 92% in the sixth pattern.

In addition, this algorithm incorporates dispersion information as a supplement. In this study, those with over-dispersed visits, defined as no identifiable COC provider, were mostly (99.7%) classified as having fewer than 9 times of PNC, with an average and median number of PNC visits of 3. A previous study demonstrated that dispersion increases as the number of chronic conditions increases [10]. If that is also the case for this study, then these persons represent a high-risk group. However, the proposed algorithm in this study, as well as previous algorithms, failed to identify the predominant provider for them. Future studies may explore alternative rules to identify the predominant provider for this group.

Second, the identified predominant providers can be applied to better understand how patients seek PNC, particularly for pregnancy complications. In this study, pregnant individuals bypassed the nearest provider and traveled on average 5 miles further to their predominant provider. Understanding why patients might choose to receive care that creates a larger travel burden is important, as these burdens negatively impact access [22]. It is also important to understand why pregnant individuals sought care from such a large variety of providers for their PNC.

Third, such an algorithm provides a useful tool for healthcare providers and policymakers alike to enhance resources in underserved areas. By having a richer understanding of where pregnant individuals seek care, where those providers are located and why patients bypass the nearest provider, it is necessary to ensure resources are allocated to maximize accessibility. Once this is better understood, efforts to increase PNC providers in specific areas or provide training to existing providers would be more effective. The underlying reasons for a provider to become a predominant provider or not can inform tailored interventions. For example, if no predominant provider can be identified for a patient due to too few PNC visits. The intervention should focus on improving PNC utilization. Furthermore, recent evidence showed that improved continuity of care during pregnancy is associated with better mental health [25] and birth outcomes [26]. Continuity of care may be enhanced with the information provided by the predominant provider analysis. If no predominant provider can be identified for a patient due to pregnancy complications and switching to another provider, close supervision of pregnancy and coordination between providers can improve patients’ experience and outcomes through improved information or relationship continuity [27].

Future studies are needed to validate this algorithm with direct interviews of pregnant individuals or electronic health records to verify their predominant provider. The validity of different patterns should also be confirmed. For example, researchers may plan to include the most visited provider with the last visit in the predominant providers to detect the referral relationship between PNC providers and delivery hospitals. They should recognize that the referral relationship in claims data was not confirmed. The consistency of these two referral relationships should be verified. These interviews should also inquire about why patients seek care with providers who are further away, accepting additional travel burdens.

The proposed algorithm can only be applied in PNC settings because the sequential information adopted in the algorithm was guided by the APNCU, which gives a high weight to the first visit [16]. Therefore, this algorithm may not be applicable in other care settings or for pregnant individuals in different contexts. Although this study applied the algorithm exclusively to South Carolina Medicaid beneficiaries, future study can apply it to claims from other state payors. A unique feature of South Carolina Medicaid is the SCRFA assigned provider identifier, which can be ascribed at a facility or a person level, whichever is applicable. That is the reason why this study constructed a specialty-specified provider identifier. However, multiple professionals with identical specialties in the same facility cannot be identified separately, which may lead to an overestimated probability of predominant providers in this study. Data acquired from other sources with a provider identifier at the professional level, such as the National Provider Identifier, would alleviate this burden for researchers and provide more reliable results.

Another limitation of using claims data to identify a predominant provider is that both frequency and sequence information require complete visit information throughout the study period, in this case, the pregnancy. This study addressed this by excluding enrollees without continuous coverage. Therefore, the results of this study can only be applied to those with continuous coverage.

Future studies may use data from all payors to identify a predominant provider. However, Medicaid enrollees differ from enrollees of other payors. Because fewer phsycians, including obstetrics and gynecologists, accept Medicaid as payment than private insurances [28], Medicaid enrollees face more barriers to accessing healthcare than those covered by private insurance [29]. The association between these access issues and the identification of a predominant provider remains unknown. A recent study reported that compared with marketplace enrollees, Medicaid enrolless were more likely to have adequate prenatal care [30]. If that were the case, visit frequency information may be more important than the sequential information in identifying a predominant provider for marketplace enrollees, according to the results of this study.

## Conclusion

By adding PNC sequential information in the algorithm, the proportion of identifiable predominant providers has increased from 81 to 92%, an increase of 11%. Different patterns covered in this algorithm provide researchers and policymakers flexibility in identifying the predominant PNC providers. Applying this algorithm reveals a longer distance for pregnant individuals travelling to their predominant PNC provider than to the nearest PNC provider.

### Supplementary Information

Supplementary Material 1.

## Abbreviations

COC: Continuity of care
PNC: Prenatal care
APNCU: Adequacy of prenatal care utilization
SC: South Carolina
RFA: Revenue and Fiscal Affair Office
DOB: Date of birth
ICD: International Classification of Diseases
CM: Clnical modification
HCPCS: Healthcare common procedure coding system
CPT: Current procedural terminology
FQHC: Federal qualified health clinic
RHC: Rural health clinic
MultPlur: Multiple providers sharing plurality
MultPlur_Initial: Multiple providers sharing plurality who conducts the first visit
MultPlur_Final: Multiple providers sharing plurality who conducts the last visit
MultPlur_InitialFinal: Multiple providers sharing plurality who conducts the first and last visits
