# Supplementary material for: Who is your prenatal care provider? An algorithm to identify the predominant prenatal care provider with claims data
Source: BMC Health Serv Res. 2024 May 27;24:665. doi: 10.1186/s12913-024-11080-2 (PMC11131320; doi:10.1186/s12913-024-11080-2)
Supplement: Supplementary file 1 — Supplementary Material 1. [file 12913_2024_11080_MOESM1_ESM.docx]

Appendix Table 1, Diagnosis and procedure codes for prenatal care identification.

| Prenatal care identification | Primary diagnosis code | | Procedure code | |
| --- | --- | --- | --- | --- |
|  | ICD-9-CM | ICD-10-CM | CPT | HCPCS |
| CPT / HCPCS alone |  |  | 59400, 59425, 59426, 59510, 59610, 59618, 99500, 0500F, 0501F, 0502F. | H1000, H1001, H1002, H1003, H1004, H1005. |
| CPT / HCPCS and ICD | V22, V23, V283, V7274. | O09, Z3201, Z32, Z363. | 99201-99205, 99211-99215, 99241-99245,98966-98972, 99421-99423, 99441-99443. | G0071, G0463, G2010, G2012, G2061-G2063, T1015. |

International Classification of Diseases: ICD; Clinical Modification: CM.

Healthcare Common Procedure Coding System: HCPCS; Current Procedural Terminology: CPT.

These codes were applied to specialty codes: 02=ALLERGY AND IMMUNOLOGY; 06=MIDWIFE; 10=EMERGENCY MEDICINE; 12=FAMILY PRACTICE; 14=GENERAL PRACTICE; 16=GYNECOLOGY; 19=INTERNAL MEDICINE; 26=OBSTETRICS; 27=OBSTETRICS & GYNECOLOGY; 40=PEDIATRICS; 48=PSYCHIATRY; 50=FEDERALLY QUALIFIED HEALTH CLINICS; 57=RHEUMATOLOGY; 78=MULTIPLE SPECIALTY GROUP; 86=NURSE PRACTITIONER; 94=DIABETES EDUCATOR; 95=DEVELOPMENTAL REHABILITATION; 97=RURAL HEALTH CLINICS (RHC); PA=PHYSICIAN ASSISTANT.

Appendix Table 2, definition of scenarios and algorithms

| # | Provider types | PNC information | Algorithm |
| --- | --- | --- | --- |
| 1 | Only | Fraction | Majority |
| 2 | Only & Majority | Fraction | Majority |
| 3 | Only, Majority & Plurality | Fraction | Plurality |
| 4 | Only, Majority, Plurality & MultPlur_Initial | Fraction & sequence | PNC version |
| 5 | Only, Majority, Plurality & MultPlur_Final | Fraction & sequence | PNC version |
| 6 | Only, Majority, Plurality, MultPlur_Initial & MultPlur_Final | Fraction & sequence | PNC version |

Note:${Fraction}_{i}=\frac{n_{i}}{N}$

N is the total number of PNC visits, and $n_{i}$ is the number of PNC visits for the *i*th provider. Only: the exclusive PNC provider for one pregnancy. Majority: this provider served more than half of all visits for one pregnancy. Plurality: the uniquely most frequently visited provider, who is the only one who served the most visits for a patient. MultPlur: most frequently visited provider, who served the most visits for a patient. MultPlur_Initial: most frequently visited provider, who served the most visits and the first visit for a patient. MultPlur_Final: most frequently visited provider, who served the most visits and the last visit for a patient.
